# Introduction

**DOI:** 10.1155/S1463924691000093

**Published:** 1991

**Authors:** Pierangelo Bonini


					Journal of Automatic Chemistry, Vol. 13, No. 2 (March-April 1991), p. 43
Special Issue: Proceedings of the
Symposium on Understanding Analytical
Systems 2: Automation of Non-isotopic
Immunoassays, San Francisco 24July 1990
In 1987 some members of the IFCC Experts Panel on Instrumentation organized a workshop on
'Selective and Random Access Analyzers', at the 13th International Congress ofClinical Chemistry
in The Hague with the sponsorship ofTechnicon International. The aim ofthat meeting was mainly
an educative one: the intention of the organizers was to help scientists, and particularly young
scientists, to understand the structure and the operation of these analysers.
The workshop was a very successful one; so it was decided to continue with them. The IFCC
Committee on Analytical Systems (the new name for the Experts Panel on Instrumentation) chaired
by Professor A. Truchaud enthusiastically endorsed this project; and Bayer Diagnostics was very
glad to sponsor it. It was held at the 14th IFCC International Congress in San Francisco; the
symposium was called 'Automation of Non-isotopic Immunoassays' and was part of a meeting on
'Understanding Analytical Systems'.
The meeting was well received by its audience and the organizers are confident that it will be
valuable to Journal ofAutomatic Chemistry's readers.
CONTENTS
Detection, signal processing and calibration in immunoassay systems, P. A. Bonini, G. BanJi,
M. Pazzagli, G. Messeri and A. Roda, p. 45.
Automated separation for heterogeneous immunoassays, A. Truchaud, ,]. Barclay, .P. Yvert and
B. Capolaghi, p. 49.
Specific requirements for automation of immunoassays in 1990,.R. Haeckel, p. 53.
Sample preparation and processing, T. D. Geary, p. 57.
PIERANGELO BONINI
43

				

